# Increasing photosynthetic benefit with decreasing irrigation frequency in an Australian temperate pasture exposed to elevated carbon dioxide

**DOI:** 10.1093/jxb/erae511

**Published:** 2025-04-09

**Authors:** Melika L Missen, Martin G De Kauwe, Mark J Hovenden

**Affiliations:** Biological Sciences, School of Natural Sciences, University of Tasmania, Hobart, Tasmania 7001, Australia; School of Biological Sciences, University of Bristol, Bristol BS8 1TQ, UK; Biological Sciences, School of Natural Sciences, University of Tasmania, Hobart, Tasmania 7001, Australia; Lancaster University, UK

**Keywords:** Climate change, elevated CO_2_, *Lolium perenne*, photosynthesis, soil water content, stomatal conductance

## Abstract

Elevated atmospheric CO_2_ (e[CO_2_]) often enhances plant photosynthesis and improves water status. However, the effects of e[CO_2_] vary significantly and are believed to be influenced by water availability. With a future warmer climate expected to increase the frequency and severity of extreme rainfall, the response of plants to e[CO_2_] under changing precipitation patterns remains uncertain. We examined the effects of e[CO_2_] and different irrigation regimes on perennial ryegrass in a free-air CO_2_ enrichment (FACE) experiment. Immediately after irrigation, the mean net photosynthetic rate was 21.2% higher under e[CO_2_] compared with ambient conditions. This benefit increased over time, reaching a 31.3% higher rate as days since watering increased, indicating a substantial increase in photosynthetic benefit with longer intervals between watering. Mean stomatal conductance was 21% lower in ryegrass under e[CO_2_] immediately after irrigation compared with ambient plots. However, the reduction in stomatal conductance under e[CO_2_] decreased as the interval between irrigation events increased, showing no difference 7–10 d after an irrigation event. These results imply that plants benefit most from carbon fertilization, assimilating relatively more carbon and losing less water, during periods with less frequent rainfall. These findings have significant implications for understanding leaf-level responses to climate change.

## Teaser

In a FACE experiment, e[CO_2_ ] enhanced photosynthesis in ryegrass, and this difference increased with days since watering. Conversely, the reduction in stomatal conductance under e[CO_2_ ] declined with days since watering, with implications for soil moisture depletion.

## Introduction

Anthropogenic climate change is predicted to alter rainfall patterns, as a warmer atmosphere can hold greater amounts of water vapour (Seneviratne *et al.*, 2021), implying an intensification of the global hydrological cycle. Climate models project a 2% increase in precipitation for every 1 °C of warming (Held and Soden, 2006), with changes in the frequency and intensity of precipitation events (Sillmann *et al.*, 2013). At the same time, regional changes in precipitation are linked to local drivers and future changes in the incidence of drought (Dai, 2013; Ukkola *et al.*, 2020). Climate records suggest that interannual rainfall patterns have already shifted (Hughes, 2003), and changes in seasonal rainfall patterns have led to altered vegetation trends (Bowman *et al.*, 2001). Since grasslands are typically unable to access deep water resources or groundwater, their productivity is strongly linked to precipitation (Weaver and Albertson, 1943; Nippert and Knapp, 2007). Some evidence exists that suggests a change in rainfall timing (i.e. longer periods between rainfall events) may have greater effects on plant responses than would be expected based on a change in the overall quantity of rainfall (Harper *et al.*, 2005). This is because observations indicate that rainfall variability can increase periods of water limitation in grasslands, which may, in turn, result in a grassland being more sensitive to interannual variation (Huxman *et al.*, 2004). Thus, the size and timing of precipitation events are likely to strongly interact with other key drivers of change in plant function and structure, such as the rising concentration of atmospheric carbon dioxide [CO_2_]. However, the effects of short-term fluctuations in precipitation on plant structure and function have not been investigated to the same degree as annual or seasonal precipitation (Hovenden *et al*., 2019). With atmospheric [CO_2_] projected to reach 550 ppm by the middle of the century (Carter *et al.*, 2007), investigating how elevated atmospheric [CO_2_] (e[CO_2_]) and changing precipitation patterns influence plant physiology is necessary for predicting plant responses under future global change scenarios.

The identified mechanisms underlying plant growth responses to e[CO_2_] are primarily related to two processes. First, e[CO_2_] directly stimulates net carbon assimilation by increasing carboxylation and suppressing photorespiration rates (Farquhar, 1997; Long *et al.*, 2004). Second, e[CO_2_] causes a decrease in stomatal conductance (*g*_s_) by reducing stomatal aperture (Farquhar, 1997; Medlyn *et al.*, 2001; Long *et al.*, 2004). Reductions in stomatal aperture under e[CO_2_] can lead to reduced ecosystem-scale transpiration (Ellsworth, 1999) thereby increasing soil water content (SWC). This process, known as the ‘water saving effect’ (Duursma and Medlyn, 2012), has been observed in various ecosystems, including grasslands (Morgan *et al.*, 2004), and is expected to delay the onset of drought. However, some evidence suggests that while e[CO_2_] may initially slow the rate of soil drying, this effect may not persist over the long term (Parvin *et al.*, 2019). Reduced *g*_s_ and increased net photosynthesis (*A*_net_) under e[CO_2_] induce increases in intrinsic water use efficiency (iWUE; Drake *et al.*, 1997), defined here as the ratio of the net photosynthetic rate to stomatal conductance for water vapour. The enhancement of iWUE induced by e[CO_2_] shows a clear pattern at the leaf scale; however, this response becomes more complex and variable at larger scales due to interactions between leaves, light interception, and microclimatic conditions. Some researchers argue that the iWUE response to e[CO_2_] is consistent across scales (Medlyn *et al.*, 2001; Ainsworth and Rogers, 2007), with physiological mechanisms driving changes from leaf to global levels. Others believe that scale-dependent processes, such as feedbacks and interactions within ecosystems, significantly influence the magnitude of iWUE enhancement (Leakey *et al.*, 2009; Knauer *et al.*, 2017).

Another potential mechanism underlying the interaction between e[CO_2_] and water availability is known as the ‘low C_i_ effect’ (Duursma and Medlyn, 2012). Stomatal closure, due to water stress (Chaves *et al.*, 2002), significantly restricts plant CO_2_ assimilation and is known as stomatal limitation (Jones, 1985). Stomatal limitation reduces leaf intercellular CO_2_ concentration (C_i_) and photosynthetic rates (Grassi and Magnani, 2005). When a low SWC causes a reduction of C_i_ which results in *A*_net_ operating on the steeper initial linear phase of the *A*–C_i_ curve, CO_2_ fertilization can mitigate stomatal limitations by increasing C_i_, thereby leading to a greater enhancement in photosynthesis for plants (Ellsworth *et al.*, 2012). This may lead to a more pronounced relative response of *A*_net_ to e[CO_2_] under water-limited conditions (Kelly *et al.*, 2016). The observed immediate enhancement of *A*_net_ under e[CO_2_], coupled with the ‘water saving effect’, is anticipated to amplify the relative response of biomass to e[CO_2_] during times of low soil moisture (Duursma and Medlyn, 2012; Kelly *et al.*, 2016). However, evidence for this is inconclusive, with some studies supporting it (Morgan *et al.*, 2004) and others contradicting it (Leakey *et al.*, 2009; Norby and Zak, 2011). Water deficits can also cause non-stomatal limitation of photosynthesis by directly limiting leaf biochemistry and/or decreasing mesophyll conductance (Zhou *et al.*, 2013). Changes in the ratio of *A* to C_i_ with varying soil moisture could indicate that the biochemical capacity for photosynthesis, driven by limitations in the maximum rate of carboxylation (*V*_cmax_) and maximum rate of electron transport (*J*_max_), is affected by water availability (Zhou *et al.*, 2013). These changes can alter the shape of the *A*–C_i_ curve, particularly under water limitation, though such effects can be challenging to measure directly. These uncertainties highlight the complexity of the net effect of e[CO_2_] on SWC and how this influences plant physiology, which is further complicated by a series of feedbacks (Beerling *et al.*, 1996; Centritto *et al.*, 1999; Duursma and Medlyn, 2012).

Structural acclimation by vegetation (i.e. increased leaf area) under e[CO_2_] (Leipprand and Gerten, 2006; Piao *et al.*, 2007) is widely argued to offset the reduction in transpiration (and may even increase transpiration). At the leaf scale, e[CO_2_]-induced stomatal closure could amplify the vapour pressure deficit (VPD), which may either increase the atmospheric demand for evaporation or induce further stomatal closure. Thus, species-specific reductions in transpiration are linked to the strength of stomatal control, which itself is related to the degree of coupling between the vegetation and the surrounding leaf boundary layer (Jarvis and McNaughton, 1986; De Kauwe *et al.*, 2017). Consequently, the extent of water savings and increased soil moisture due to a reduction in evapotranspiration is complex, and an improved understanding of the trade-off between *g*_s_, leaf area, and SWC under e[CO_2_] and changing precipitation frequency is necessary.

Here, we explore the effects of water availability and e[CO_2_] on physiological responses of an agriculturally important pasture species, perennial ryegrass (*Lolium perenne* L.). We did this using the TasFACE2 experimental site, the world’s only free-air CO_2_ enrichment (FACE) experiment in which soil water supply is completely controlled (Brinkhoff *et al*., 2019). To quantify precipitation variability on a pasture ecosystem, we altered temporal precipitation and size of irrigation events. By collecting high-frequency estimates of SWC, leaf gas exchange, water relations, and leaf area, we aimed to test whether precipitation timing influenced the physiological responses to e[CO_2_]. Specifically, we tested the following hypotheses: (i) e[CO_2_] will enhance WUEi (by decreasing *g*_s_ and enhancing carbon assimilation), and the difference between [CO_2_] treatments will decrease as water availability decreases (i.e. irrigation frequency decreases). These processes will lead to (ii) leaf area index (LAI) responding positively to e[CO_2_] compared with ambient conditions, and this difference will increase with decreasing irrigation frequency. As a result (iii) soil moisture will be unchanged by e[CO_2_] due to an increase in LAI.

## Materials and methods

### Experimental site description

The experiment was conducted at the TasFACE2 FACE experiment at the University of Tasmania Farm in Cambridge, Tasmania, Australia (42°48′S, 147°25′E; 50 m a.s.l). This region of Tasmania experiences a Mediterranean climate with a mean annual rainfall of 490 mm, annual mean maximum temperature of 17.6 °C, and minimum temperature of 8.2 °C [Bureau of Meteorology (BOM, 2021)]. The soils are duplex, consisting of grey-brown sandy loam to ~30 cm on sandy medium to heavy clay (Hovenden *et al*., 2023). The site was a permanent pasture for decades, supporting a community dominated by cocksfoot (*Dactylis glomerata*), perennial ryegrass (*Lolium perenne*), and white clover (*Trifolium repens*), until its establishment as a FACE experiment in 2015 (site establishment details in Brinkhoff *et al.*, 2019).

In July 2019, the site was cleared and re-sown with a monoculture of perennial ryegrass (*Lolium perenne* cv. Base AR37) at a density of 20 g seed m^–2^. Nitrogen (N), phosphorus (P), and potassium (K) were applied in rates that reduce the likelihood of nutrient limitation of growth. Three months after sowing, initial applications of N as urea and P as superphosphate were applied at a rate of 30 kg ha^–1^ and 1150 kg ha^–1^, respectively. K was applied 2 weeks later at a rate of 1000 kg ha^–1^, and subsequent applications were applied annually at 50% of the initial rate. N was applied immediately following each subsequent harvest at a rate of 2 kg N ha^–1^ d^–1^, which is the application rate at which N saturation of growth occurs in perennial ryegrass in Tasmania under well-watered conditions (Rawnsley *et al.*, 2014). P was applied at a rate of 250 kg ha^–1^ every 6 months to prevent P limitation (Gourley *et al.*, 2007). At the beginning of the first summer (December 2019), the first harvest was performed, and the biomass discarded.

The site consisted of 12 experimental ‘rings’ (1.8 m diameter, Supplementary Fig. S1) located under 3×3 m rainout shelters to minimize the influence of natural precipitation on soil moisture. e[CO_2_] was achieved through injection of pure CO_2_ as described in Hovenden *et al*. (2006). The levels of atmospheric CO_2_ were ~400 μmol mol^–1^ (ambient) and 550 μmol mol^–1^ (elevated), consistent with other experimental studies and the expected concentration for around 2050 (Smith and Myers, 2018). [CO_2_] of each elevated ring was constantly monitored by an infrared gas analyser (IRGA), and CO_2_ flow rates were adjusted by a proportional integration device algorithm (performance details in Hovenden *et al*., 2006). CO_2_ fumigation commenced each day at sunrise and ceased at sunset, with CO_2_ fumigation being carried out all year round, continuing in all weather conditions including high winds. Using a randomized split plot design, [CO_2_] treatments were replicated six times, and each ring was divided into five equal sectors, allowing the employment of five watering treatments (Supplementary Fig. S1). Importantly, each sector was isolated from its neighbour and the surrounding soil with plastic sheeting to a depth of 1 m (Brinkhoff *et al.*, 2019). Perennial ryegrass pasture has a relatively shallow root zone, with 40% of its roots found in the top 0.10 m of soil, and 80% of its roots found in the top 0.30 m of soil (Crush *et al.*, 2005).

### Irrigation frequency treatments

Temporal rainfall data were assessed to establish irrigation frequency intervals (proxies for rainfall events), which revealed relatively short durations between successive rainfall events at the site. Therefore, irrigation intervals were chosen at 3, 5, and 10 d, with the overall irrigation amount being the same over a 30 d period. All sectors received water supply that was adequate to maintain field capacity. Irrigation treatments were employed from February 2020 through to September 2021. Water was supplied by five evenly spaced drippers in each sector that were automatically controlled and independent of other sectors. Water amounts for field capacity were determined using the biophysical pasture simulation model DairyMod (Cullen *et al*., 2008). Irrigation occurred in one event between 02.00 h and 05.00 h for the 3 d and 5 d treatments, while the 10 d treatment received its water supply over two watering events to limit any run-off. For this treatment 50% of the water was delivered between 21.00 h and 24.00 h and the other 50% was delivered between 02.00 h and 05.00 h on the same night.

### Soil water content

Underground time-domain reflectometry (TDR) sensors were installed horizontally in all sectors of eight of the 12 rings (CS616; Campbell Scientific, Logan, UT, USA) at a depth of 20 cm. From these, SWC was continuously logged and recorded half hourly on a CR100 datalogger (Campbell Scientific Australia Pty Ltd, Townsville, QLD, Australia).

### Gas exchange

Measurements of net CO_2_ assimilation (*A*_net_; µmol CO_2_ m^−2^ s^−1^) and stomatal conductance (*g*_s_; mol H_2_O m^−2^ s^−1^) were conducted on ryegrass at growth [CO_2_] using the Li-Cor LI6400 and LI6400XT portable photosynthesis systems (Li-Cor, Inc., Lincoln, NE, USA). Fully developed young leaves in good condition were measured throughout the morning and early afternoon (08.00 h and 14.00 h), in line with best practice and to avoid the possibility of afternoon stomatal closure, throughout 2020 and 2021. All measurements were made at a photosynthetic photon flux density of 1000 µmol photons m^−2^ s^−1^ [mean day photosynthetically acitve radiation (PAR) for the study period was 1370.1±283.6 μmol m^−2^ s^−1^], ambient temperatures, and growth [CO_2_]. Growth [CO_2_] was set via the CO_2_ injection system, with CO_2_ref set so that the CO_2_ concentration in the chamber matched the target treatment [CO_2_] concentrations (400 μmol mol^–1^ and 550 μmol mol^–1^). Relative humidity was ambient inside the leaf chamber [mean chamber VPD (VPD_leaf_: 1.26±0.03 (SE)]. Gas exchange measurements were logged every 30 s for a minimum of 10 min after leaf *g*_s_ stabilized. Five consecutive, stable data points were chosen and averaged to estimate steady-state gas exchange of each leaf.

In each irrigation treatment, sampling days were chosen to represent a range of time since watering. Measurements were conducted to capture key points just after irrigation events, as well as a range of days into the irrigation cycles. The 3 d irrigation treatment was sampled on 1, 2, and 3 d after an irrigation event, while the 5 d irrigation treatment was sampled on 1, 2, 3, and 5 d after an irrigation event (the day of an irrigation event was called day 1). The 10 d irrigation treatment was sampled on 2, 5, 7, 9, and 10 d after an irrigation event, so measurements were made on more days in sectors receiving irrigation less frequently (see Supplementary Table S1 for details on sampling times, replicates, and sample sizes). Gas exchange measurements were used to calculate iWUE (µmol mol^–1^), defined as the ratio of the fluxes of net photosynthesis (µmol m^–2^ s^–1^) and stomatal conductance to water vapour (mol m^–2^ s^–1^). This provides insight into stomatal regulation and has been described by Farquhar and Richards (1984) as:


$$\begin{array}{l} iWUE=A_{net}/g_{s} \end{array}$$

### Water potential

During summer (January to mid-March), leaves were collected pre-dawn [before stomatal opening, measured by an optical dendrometer (Bourbia *et al.*, 2022)] and at noon (when stomata were at steady state) for water potential measurements (Ψ_leaf_). The optical dendrometers were only used to evaluate when stomata were closed (for pre-dawn measurements) and when steady state was reached (for midday measurements). The youngest fully expanded leaves were sampled, covering a range of days since irrigation. Measurements were conducted on specific days after an irrigation event to capture key points in the irrigation cycles. The 3 d irrigation treatment was sampled on days 1 and 3 after an irrigation event, while the 5 d irrigation treatment was sampled on days 2, 3, 4, and 5 after an irrigation event (the day of an irrigation event was called day 1). The 10 d irrigation treatment was sampled on days 2, 3 5, 7, and 9, after an irrigation event, so samples were taken on more days in sectors receiving irrigation less frequently (see Supplementary Table S2 for details on sampling times, replicates, and sample sizes). Leaves were wrapped in moist paper towels, sealed in two zip-lock bags, and placed in a cooler, before being returned to the laboratory where Ψ_leaf_ was measured using a Scholander pressure chamber. Measurements were done within 8 h of collection.

### Leaf area index

A subsample of each harvest (~6 g FW) was weighed and scanned on a flat-bed scanner (Toshiba e-STUDIO5015AC) to quantify leaf area. All samples were then dried at 60 °C for 1 week and weighed again for dry weight. The scanned images were analysed using ImageJ (Version 1.53a, National Institute of Health, Bethesda, MD, USA) to give a leaf area (mm^2^ g^–1^ DW). Using the overall dry weight of harvested above-ground biomass, we then calculated leaf area index per metre (LAI, m^2^ m^–2^).

### Statistical analyses

To account for variation in the underlying moisture levels among plots, we expressed SWC during the experimental period relative to the mean SWC during June–August (RSWC). All plots received the same initial irrigation treatment during the winter of 2020. Plot-level RSWC was expressed by averaging the 30 min values over the duration of each watering cycle (i.e. over each 3, 5, or 10 d period) and using these values to calculate monthly mean relative SWC at the plot level. In addition, soil moisture variability was quantified using two metrics: (i) the coefficient of variation of SWC (CV_swc_), which shows variability (SD) in mean daily SWC as a percentage of SWC; and (ii) the mean change in soil moisture between successive individual irrigation events, which describes the amplitude of variation of SWC (∆SWC=SWC_max_–SWC_min_)/SWC_max_×100; Fay *et al.*, 2011).

All statistical analyses were conducted in R v.4.0.2 (R Core Team, 2021). Linear mixed-effects models with repeated measures were fit using the lme4 package (Bates *et al.*, 2015) to test the effects of [CO_2_], irrigation, and month on RSWC, ∆SWC, *A*_net_, *g*_s_, iWUE, Ψ_leaf_, and LAI. In all models, sector, nested within ring, was included as a random effect, which accounted for repeated sampling over the study period and environmental differences among plots. Stomatal conductance is strongly related to VPD. Thus, we included VPD as a covariate in our models of gas exchange to control for microclimatic conditions in rings, thus reducing confounding effects of environmental variability (*g*_s_ and *A*_net_). An ANOVA (Type II) was performed on the fitted model using the ‘Anova’ function from the car package (Fox *et al.*, 2012), specifying the test statistic as the *F*-test. Where required, we determined pairwise differences in irrigation and [CO_2_] using Tukey’s post-hoc test (emmeans R package; Lenth, 2022). To ensure the validity of our analysis, we performed tests for heteroscedasticity and normality on all data. We utilized both plotting methods and the Box–Cox function of the MASS package (Ripley *et al.*, 2013) in R for this purpose. In cases where dependent variables did not meet the requirements for these tests, appropriate transformations were performed as indicated by the Box–Cox test. RSWC and iWUE were log transformed, while ∆SWC, *g*_s_, LAI, and Ψ_leaf_ were square root transformed. The number of replicates varied between measured parameters and treatments, and is indicated in the figure legends.

It was evident that the time since an irrigation event influenced *A*_net_ and *g*_s_; however, this was difficult to quantify because of the different durations of irrigation treatments. First, we pooled all irrigation treatments and explored the change in *A*_net_ and *g*_s_ over time between [CO_2_] treatments. Linear mixed-effects models were used to test the response of *A*_net_ and *g*_s_ to the number of days since an irrigation event and how this differed between [CO_2_] treatments. Where required, we determined pairwise differences in days since watering using Tukey’s post-hoc test. Second, we binned *A*_net_ and *g*_s_ measurements based on when they were sampled in proximity to an irrigation event. These categories were defined based on observed significant differences in *g*_s_ among irrigation treatments and individual days. Notably, 1 d since irrigation was significantly different from 7 d since irrigation (*P*<0.05). Consequently, we divided the data into two categories: the first encompassed days 1–5 since an irrigation event, including measurements from all three irrigation treatments, while the second comprised days 7–10 since an irrigation event, incorporating data exclusively from the 10 d irrigation treatment (day 6 was not measured).

## Results

### Soil water content

**Fig. 1.**
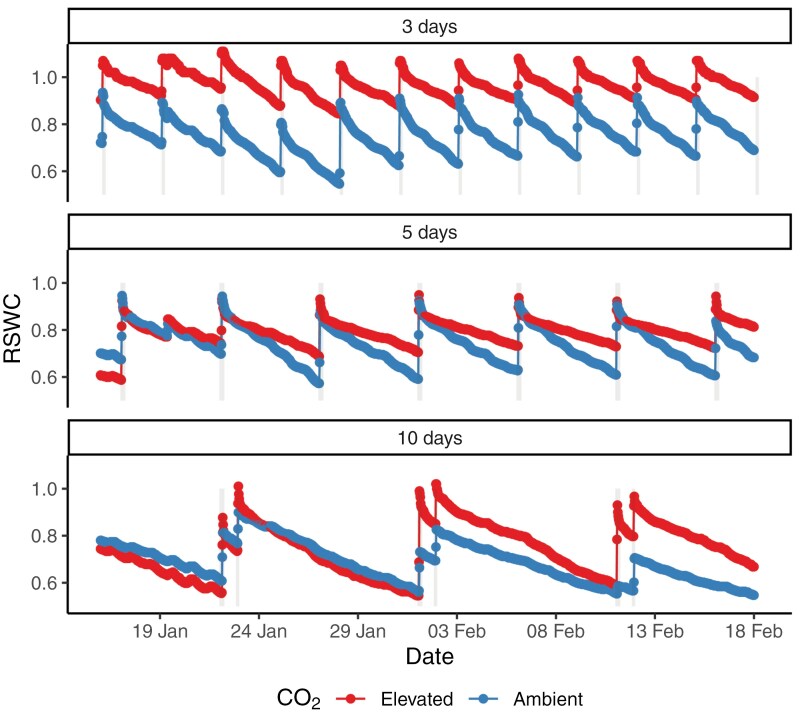
Relative soil water content (RSWC) from a representative pair of plots illustrating dry down and rewetting following the irrigation treatments (watered every 3, 5, and 10 d) under elevated and ambient [CO_2_]. The grey areas indicate days on which watering events occurred (watering for the 10 d irrigation treatment occurred over two events on the same day to reduce run-off).

**Fig. 2.**
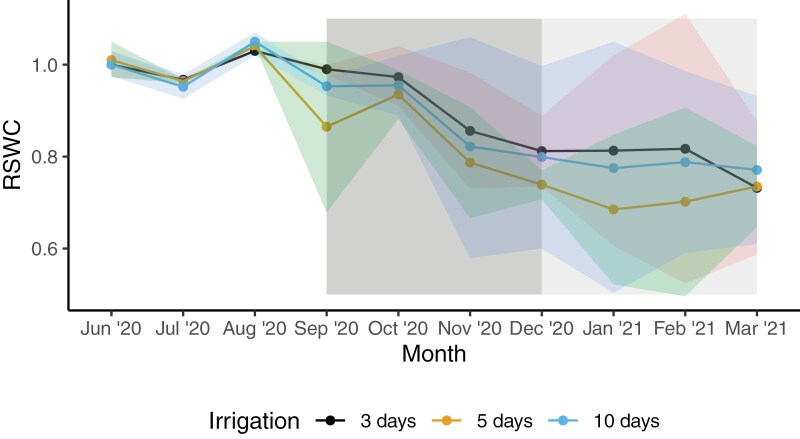
Monthly means of relative soil water content (RSWC, solid line), expressed as SWC during the experimental period relative to the mean SWC during June–August 2020. The dark grey shading is spring, the light grey shading represents the rest of the growing season (December–February), and ribbons are 95% confidence intervals. Soil water content sensors were used in all irrigation treatments in eight rings; therefore, there were four replicates of any given [CO_2_] and irrigation combination.

RSWC variation over time clearly followed irrigation patterns, with plots drying and rewetting following the irrigation schedule (Fig. 1); however, high variation within treatments made it difficult to detect irrigation or [CO_2_] effects. Thus, irrigation frequency did not alter mean monthly SWC (*f*=0.48*, P*=0.51) or mean monthly RSWC (*f*=2.00, *P*=0.18; Table 1). However, there was an irrigation×month effect on RSWC (*f*=1.96, *P*=0.015; Table 1), indicating that irrigation frequency impacted soil water availability in particular months (albeit not a persistent effect). Specifically, plots irrigated every 5 d had lower mean SWC in September compared with plots irrigated every 3 d and 10 d (*P*<0.05; Fig 2). [CO_2_] did not affect RSWC, nor did irrigation frequency interact with [CO_2_] to affect RSWC. Overall, there was some decline in RSWC over the study period (Fig. 2) and treatments did not substantially alter this seasonal pattern.

**Table 1.** Output of linear mixed-effects models reporting ANOVA on relative SWC (RSWC), amplitude of change in SWC (∆SWC), stomatal conductance (*g*_s_), net carbon assimilation (*A*_net_), intrinsic water use efficiency (iWUE), and leaf area index (LAI) as affected by irrigation regime, atmospheric CO_2_ concentration ([CO_2_]), and month, and their interactions

Variable	Irrigation F P		[CO_2_] F P		Month F P		[CO_2_]×Irrigation F P		[CO_2_]×Month F p		Irrigation×Month F P		[CO_2_]×Irrigation×Month F P	
RSWC	2.00	0.18	0.00	0.97	100.51	**<0.001**	0.31	0.74	0.90	0.53	1.96	**0.015**	0.66	0.86
∆SWC (%)	11.54	**0.002**	0.27	0.62	138.96	**<0.001**	0.35	0.71	2.20	**0.020**	1.88	**0.014**	1.37	0.14
g_s_	15.13	**0.001**	24.91	**0.005**	8.99	**<0.001**	2.07	0.18	2.63	**0.027**	2.41	**0.014**	1.06	0.39
A_net_	17.47	**0.001**	23.72	**0.002**	11.98	**<0.001**	0.14	0.87	3.24	**0.008**	3.92	**<0.001**	0.73	0.68
iWUE	3.04	0.09	134.17	**<0.001**	18.02	**<0.001**	2.66	0.12	1.71	0.14	2.10	**0.033**	0.87	0.56
LAI	1.92	0.18	0.23	0.64	28.54	**<0.001**	0.92	0.42	1.87	**0.050**	0.73	0.79	0.72	0.80

**Fig. 3.**
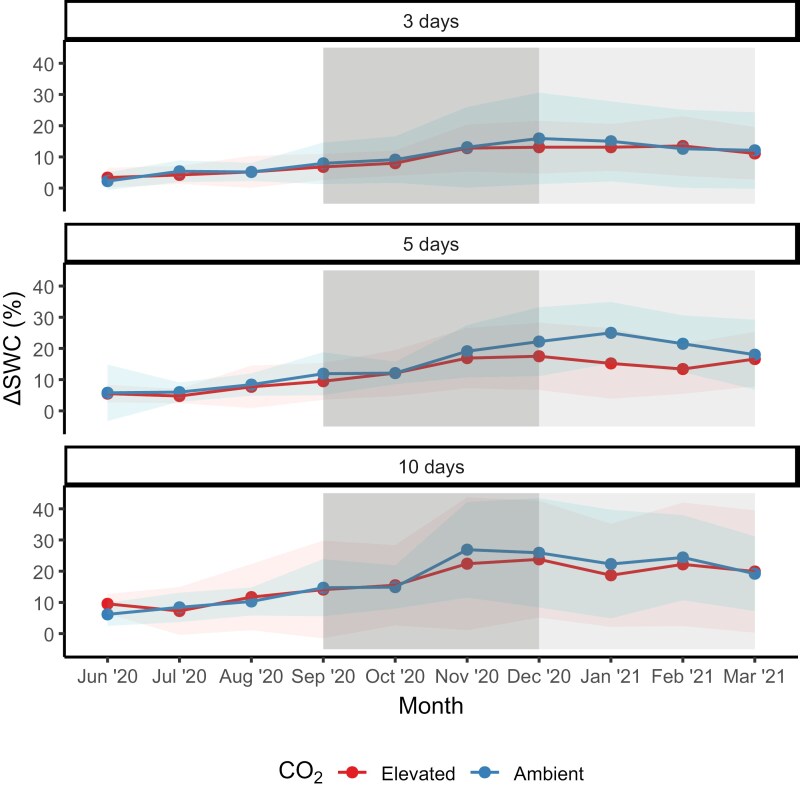
Amplitude of change in soil water content during an irrigation cycle (∆SWC, solid line) averaged for each month of the experimental period. ∆SWC was expressed as a percentage of the difference between the highest and lowest SWC within a single irrigation cycle of three irrigation treatments at elevated and ambient [CO_2_]. Ribbons are confidence intervals of four replicates of ∆SWC, dark grey shading is spring. and light grey is summer.

The irrigation treatments caused cyclic variation in SWC, measured as ∆SWC, the difference between the highest and lowest SWC within a single irrigation cycle as a percentage. There was a [CO_2_]×month interaction (*f*=2.20; *P*=0.020, Table 1) on ∆SWC (Fig. 3); however, post-hoc tests did not detect differences between ambient and e[CO_2_] plots in any months. There was also an irrigation×month interaction (*f*=1.88, *P*=0.01), because ∆SWC was significantly different in some irrigation treatments compared with others in some months. Specifically, plots watered every 3 d had significantly lower ∆SWC than plots watered every 10 d in all months (*P*<0.05), except July 2020. Furthermore, plots watered every 3 d had significantly lower ∆SWC than plots watered every 5 d in November 2020, December 2020, January 2021, and March 2021 (*P*<0.05). This suggests that there were slight differences in the depletion in soil moisture amongst irrigation and [CO_2_] treatments; however, high variation within treatments obscured these signals, and [CO_2_]×irrigation×month was non-significant (*f*=1.37, *P*=0.139).

### Gas exchange

**Fig. 4.**
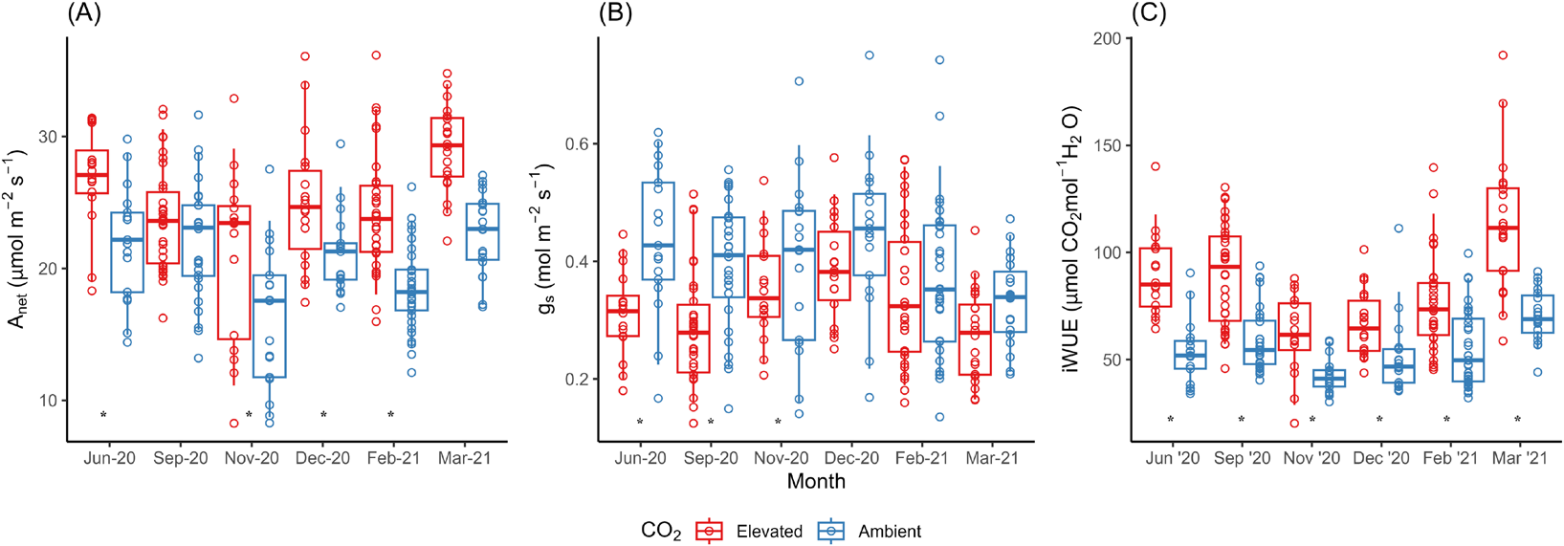
Monthly photosynthetic traits of perennial ryegrass under e[CO_2_]. Monthly mean net carbon assimilation rate (A; *A*_net_), stomatal conductance (B; *g*_s_), and intrinsic water use efficiency (C; iWUE) measured in plants of ryegrass at ambient (blue, 400 ppm) and elevated [CO_2_] (red, 550 ppm). Boxes represent the mean and first and third quartile values for each treatment combination, and symbols represent individual measurements in 30 sectors with 6–17 repeated measures depending on treatment (see Supplementary Table S1; an asterisk indicates a significant difference between [CO_2_] treatments in a given month).

**Fig. 5.**
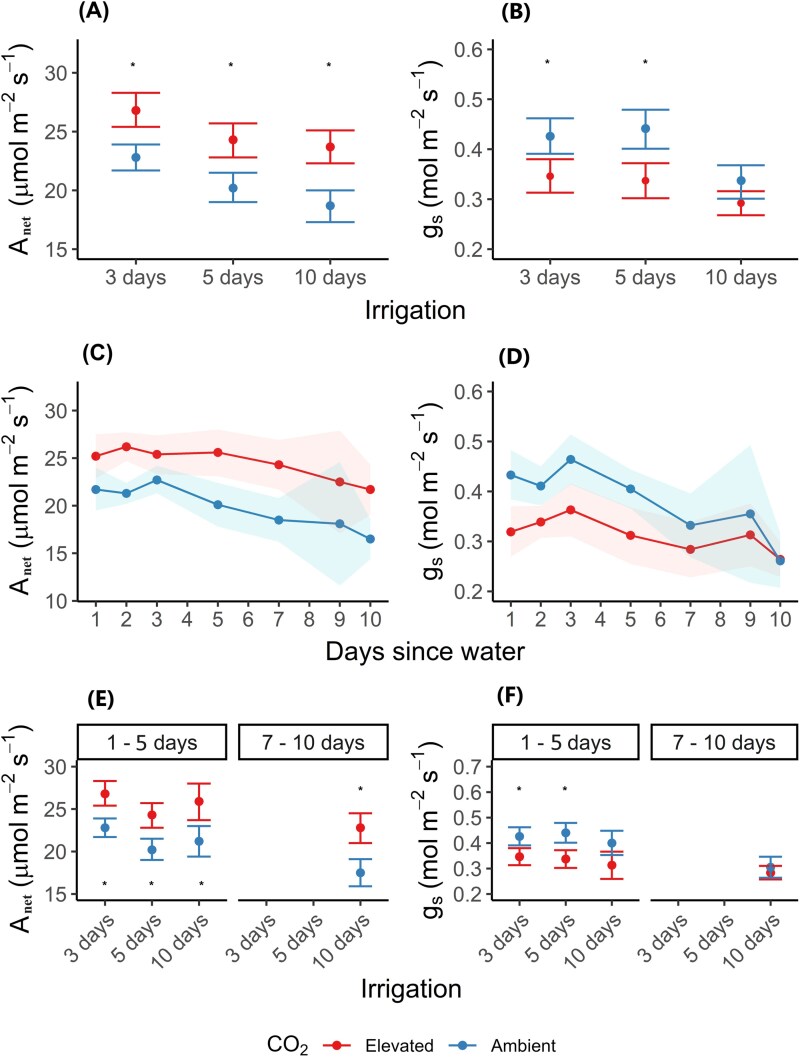
Photosynthetic traits across irrigation treatments. Mean net carbon assimilation rate (*A*_net_) and mean stomatal conductance (*g*_s_) of each irrigation treatment over all days of their irrigation cycles (A) and (B). Mean *A*_net_ and *g*_s_ of each day since watering event with all irrigation treatments pooled (C) and (D), and days since watering pooled into two categories (E) and (F). Data represent the mean ±confidence intervals, and an asterisk indicates significant difference between [CO_2_] treatments.

We measured net carbon assimilation (*A*_net_) under growth conditions (i.e. plants under e[CO_2_] were measured at 550 µmol mol^−1^ and plants under ambient [CO_2_] were measured at 400 µmol mol^−1^). A full and comprehensive assessment of the effectiveness of the fumigation treatments and temporal and spatial variability using this experimental infrastructure is provided in Hovenden et al. (2006). Long-term mean [CO_2_] in FACE rings was 549±0.1 ppm, with the [CO_2_] being within 20% of the set-point for 97% of the time and within 10% of the set-point for 85% of the time (Hovenden et al., 2006). The overall enhancement of mean leaf *A*_net_ under e[CO_2_] was 22% (24.86±0.43 μmol CO_2_ m^–2^ s^–1^; *f*=23.72, *P*=0.002) compared with ambient plots (20.38±0.39 μmol CO_2_ m^–2^ s^–1^; *f*=23.72, *P*=0.002). However, this differed monthly as shown by the significant [CO_2_]×month interaction because e[CO_2_] enhanced *A*_net_ in some months and not others (*f*=3.24, *P*=0.008; Fig. 4A). Specifically, *A*_net_ was significantly higher in plants under e[CO_2_] compared with plants in ambient plots in all months (*P*<0.05) except for September (*P*=0.24). There was also an irrigation×month effect on *A*_net_ (*f*=3.92, *P*<0.001) because plants in the 10 d irrigation treatment had significantly lower *A*_net_ in November compared with plants in the 3 d and 5 d treatments (*P*<0.05). In addition, plants irrigated every 5 d and 10 d had significantly lower *A*_net_ than plants irrigated every 3 d in December 2020 and February 2021 (*P*<0.05). There was also an irrigation effect on *A*_net_ (*f*=17.47, *P*<0.001; Fig. 5A), because, overall, plants irrigated every 10 d had significantly lower *A*_net_ than plants irrigated every 3 d.

The overall reduction of mean *g*_s_ under e[CO_2_] was 18% (0.32±0.01 mol H_2_O m^–2^ s^–1^; *f*=24.91, *P*=0.005) compared with ambient plots (0.39±0.01 mol H_2_O m^–2^ s^–1^; *f*=24.91, *P*=0.005). There was a CO_2_×month interaction on mean *g*_s_ (*f*=2.63, *P*=0.03), and in June, September, and November mean *g*_s_ was significantly lower in plants in e[CO_2_] plots compared with ambient plots (*P*<0.05; Fig. 4B). Irrigation frequency (*f*=15.13, *P*=0.001; Fig. 5B) strongly influenced overall mean *g*_s_; however, it was clear that this influence was dependent upon the number of days since an irrigation event; that is, measurements taken on the day following irrigation were significantly different from measurements taken 10 d after an irrigation event. Thus, in the days immediately following irrigation, both *A*_net_ and *g*_s_ of plants within [CO_2_] treatments were similar across the irrigation treatments; that is, plants irrigated every 3 d under ambient [CO_2_] were not significantly different from plants irrigated every 5 d and 10 d under ambient [CO_2_] (*P*>0.05). However, as the length of time since irrigation increased, the [CO_2_] effects on *A*_net_ and *g*_s_ became more pronounced (Fig. 5C, D).

As days since irrigation increased, *A*_net_ generally declined overall; however, the stimulation of photosynthesis by e[CO_2_] was maintained in ryegrass plants over the entire irrigation cycle (Fig. 5C). This was due to [CO_2_] (*f*=7.72, *P*=0.004) and days since irrigation (*f*=4.13, *P*<0.001) having significant effects on *A*_net_; however, there was no interaction between these two factors (*f*=167.8, *P*=0.80). Directly after irrigation, *A*_net_ of ryegrass plants was 21.2% higher in e[CO_2_] plots (25.91±0.08 μmol CO_2_ m^–2^ s^–1^) than that of plants in ambient plots (21.38±0.07 μmol CO_2_ m^–2^ s^–1^; *P*<0.05). Importantly, e[CO_2_] stimulated *A*_net_ to a greater extent (31.3%) 10 d after irrigation than in days earlier during the irrigation cycle, even though *A*_net_ tended to decrease over time in all plots (*P*<0.05). By the end of the irrigation cycle 10 d after irrigation, mean *A*_net_ of plants growing at e[CO_2_] was 21.68±0.31 μmol CO_2_ m^–2^ s^–1^ while that of plants in ambient plots was 16.51±0.97 μmol CO_2_ m^–2^ s^–1^. The general decline in RSWC (Fig. 1) with increasing time since irrigation was accompanied by a reduction is *g*_s_ over time (days since water effect; *f*=52.33, *P*<0.001) (Fig. 5D); however, this reduction was much greater in ambient plots than in e[CO_2_] plots because *g*_s_ was initially higher in plants growing at ambient [CO_2_] (Fig. 5D). Thus, immediately after irrigation, mean *g*_s_ of ryegrass plants in e[CO_2_] plots (0.33±0.002 mol H_2_O m^–2^ s^–1^) was 21% lower than in ambient plots (0.42±0.002 mol H_2_O m^–2^ s^–1^; *P*<0.05). This difference in *g*_s_ between plants growing at ambient and e[CO_2_] declined relatively steadily over time and was negligible 10 d after irrigation (e[CO_2_], 0.26±0.02 mol H_2_O m^–2^ s^–1^; ambient, 0.26±0.03 mol H_2_O m^–2^ s^–1^; Fig. 5D; *P*=0.80).

To further investigate this dependency of the CO_2_ effect on days since irrigation, we split *g*_s_ and *A*_net_ into 1–5 d and 7–10 d since the last irrigation event (no data were collected for 6 d after irrigation). We chose this distinction because the 3 d and 5 d irrigation treatments showed no difference in mean *g*_s_ but were significantly different from the 10 d irrigation treatment (*f*=15.13, *P*=0.001). At 1–5 d since watering, stomatal conductance was reduced in plants under e[CO_2_] compared with ambient [CO_2_]; however, at 7–10 d after watering, *g*_s_ was similar between [CO_2_] treatments, as seen by a significant [CO_2_]×days since watering, interaction (*f*=6.12, *P*=0.01; Fig. 5F). *A*_net_ was enhanced in plants under e[CO_2_] compared with ambient [CO_2_] at both 1–5 d and 7–10 d since watering (*f*=16.23, *P*=0.004), although there was some decline in both the control and e[CO_2_] treatments at 7–10 d (*f*=18.25, *P*=0.001).

### Water relations

The results of the gas exchange measurements demonstrated a linear and significant correlation between *g*_s_ and *A*_net_ (*f*=43.813, *P*<0.0001, *R*^2^=48.28). iWUE was 50.1% higher at e[CO_2_] compared with ambient plots over all recorded months (*f*=134.17, *P*=0.0002; Table 1; Fig. 4C); however, irrigation frequency did not influence iWUE (*f*=3.04, *P*=0.09; Table 1). iWUE peaked in March 2021 under e[CO_2_] (113.82±7.12 μmol CO_2_ mol^–1^ H_2_O) which coincided with one of the drier months recorded; however, iWUE was also high under e[CO_2_] in wet months such as June 2020 (89.62±4.90 μmol CO_2_ mol^–1^ H_2_O).

**Fig. 6.**
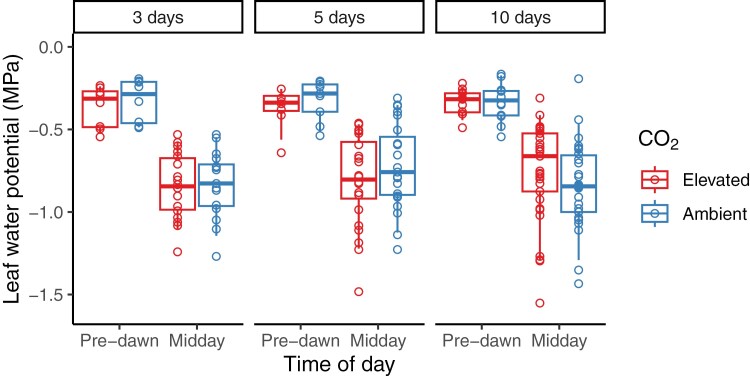
Pre-dawn and midday leaf water potential (Ψ_leaf_) of ryegrass during summer at elevated and ambient [CO_2_]. Boxes represent the mean and first and third quartile values for each treatment combination, and symbols represent individual samples in 24 sectors with 8–33 repeated measures depending on treatment (see Supplementary Table S2).

The water potential of the youngest fully expanded leaves was determined from pre-dawn and midday leaf samples during the summer months. Overall, mean Ψ_leaf_ did not respond to [CO_2_] (pre-dawn, *f*=0.85, *P*=0.41; midday, *f*=0.15, *P*=0.74) or irrigation treatments (pre-dawn, *f*=0.03, *P*=0.97; midday, *f*=0.94, *P*=0.39) at pre-dawn or midday (Fig. 6). Days since the last irrigation event also had no effect on Ψ_leaf_ at pre-dawn (*f*=2.17, *P*=0.06) or midday (*f*=0.99, *P*=0.44), suggesting that there was no plant water status pattern over time. Our results suggest that [CO_2_] did not affect plant water status, with Ψ_leaf_ highly variable across replicates. To determine whether mean SWC determined Ψ_leaf_, the response of both pre-dawn and midday Ψ_leaf_ to mean SWC was examined, but neither relationship (pre-dawn Ψ_leaf_, *f*=1.48, *P*=0.22; midday Ψ_leaf_, *f*=1.88, *P*=0.19) was significant.

### Leaf area index

**Fig. 7.**
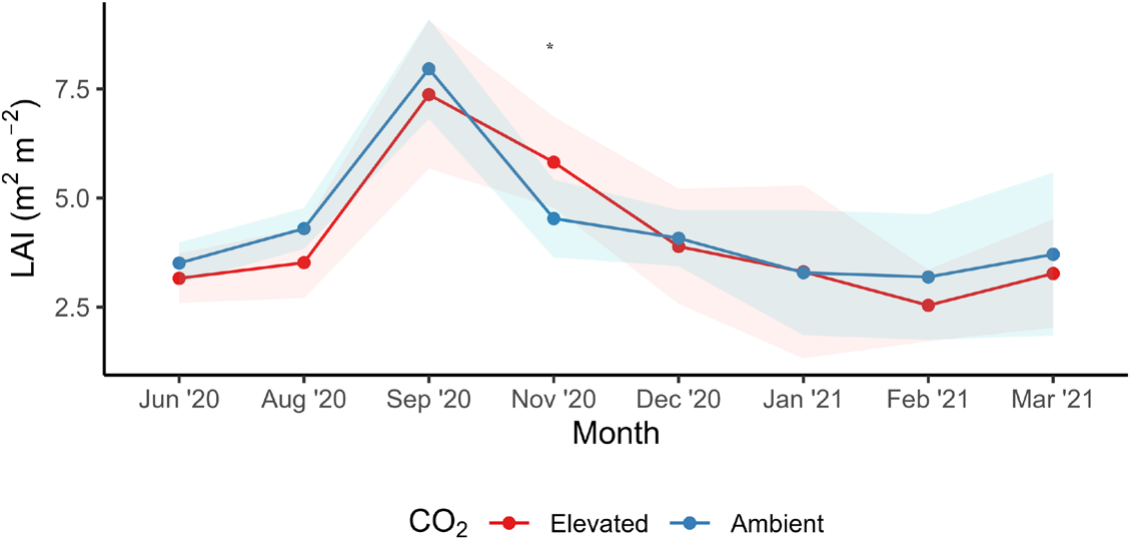
Mean leaf area index (LAI) for the study period from June 2020 to March 2021. Solid lines indicate means of six replicates averaged across three irrigation treatments, and dashed lines represent 95% confidence intervals (* indicates significant difference between [CO_2_] treatments in a given month).

LAI was highest during the growing season, namely in September to November. There was a significant [CO_2_]×month interaction on mean LAI (*f*=1.87, *P*=0.05; Table 1; Fig. 7). This was due to no differences in LAI in plants grown under e[CO_2_] compared with control plots in all months except for November (Fig. 7). In November 2020, LAI values of plants under e[CO_2_] were 29% higher compared with ambient plots (*P*=0.023). Mean LAI also showed no statistically significant response to irrigation (*f*=1.92, *P*=0.17).

## Discussion

Interannual variability in rainfall can make it difficult to detect changes in plant physiology in response to e[CO_2_], particularly when variation in SWC obscures the ability to discriminate e[CO_2_] effects (Gray *et al.*, 2016). Studies often do not assess differences among years in soil moisture depletion, and observed changes to SWC may be dominated by background rainfall variability (De Kauwe *et al.*, 2021). Many studies that try to assess the CO_2_ effect on soil water content often encounter such widely varying precipitation in the field that they cannot disentangle the response to [CO_2_] from the response to rainfall. Thus, previous field studies have reported increased (Lecain *et al.*, 2003), decreased (Samarakoon and Gifford, 1995), or unaffected SWC (Kellner *et al.*, 2019) in response to e[CO_2_]. Furthermore, multi-year studies have reported that retention of SWC due to e[CO_2_] is not consistently observed from year to year (Morgan *et al.*, 2011; Gray *et al.*, 2016), showing the strong context dependency of what is often considered a simple consequence of exposure to e[CO_2_] (Wang *et al.*, 2022). To tackle this issue, our study attempts to control for precipitation and investigate the underlying physiological responses to e[CO_2_]. Our results demonstrated that net carbon assimilation was enhanced under e[CO_2_] by 21.2% immediately after irrigation, and this stimulation increased as days since irrigation increased (to 31.3% 10 d after irrigation). Plants under e[CO_2_] experienced a reduction in *g*_s_ 1–5 d after irrigation, after which (7–10 d after irrigation) stomatal closure in control plants was similar to that of plants under ambient [CO_2_]. The observed increase in *A*_net_ led to an increase in LAI early in the growing season (November); however, for the rest of the year, LAI was unchanged by e[CO_2_]. These physiological responses may have led to a biological but not statistically significant reduction in ∆SWC under e[CO_2_] compared with control plots in the 5 d irrigation treatment during the summer (Fig. 2). If this were the case, e[CO_2_] may have stimulated soil moisture benefits in plots irrigated every 5 d, due to the sustained reduction in *g*_s_. These results suggest that physiological mechanisms of perennial ryegrass are sensitive to relatively small changes in [CO_2_] and irrigation frequency, with implications for future pasture productivity.

### Treatment impacts on gas exchange and water use efficiency

Previous studies have reported increases in *A*_net_ of 13–46% in ryegrass under e[CO_2_] between 550 ppm and 600 ppm (Rogers *et al.*, 1998; Isopp *et al.*, 2000; Kimball *et al.*, 2002; Ainsworth *et al.*, 2003; Yiotis *et al.*, 2021), and other grass species have shown greater *A*_net_ stimulation under reduced irrigation amounts (Volk *et al.*, 2000). Similarly, we found that *A*_net_ was stimulated under e[CO_2_] relative to control plots in all irrigation treatments and for the duration of the irrigation cycles. In fact, *A*_net_ was stimulated to a greater extent (i.e. 31.3%) 10 d after an irrigation event than immediately after irrigation (i.e. 21.2%), despite *A*_net_ decreasing over time in all plots (Fig. 5D). This difference is consistent with theoretical enhancement of photosynthesis under e[CO_2_], and is attributed to the enzyme Rubisco, which is not CO_2_ saturated at present [CO_2_] (Long, 1991). The stimulation of *A*_net_ in ryegrass under decreasing irrigation frequency suggests that plants under e[CO_2_] can maintain *A*_net_ for longer than plants under ambient [CO_2_]. This may be attributed to the greater decline in *g*_s_ in ambient plants between the start and end of the 10 d irrigation cycle compared with FACE plants (Fig. 2). Thus, plants in control plots reduced their stomatal aperture towards the end of the 10 d cycle, resulting in a decrease in *A*_net_, leading to a greater difference between plants under elevated and ambient [CO_2_]. This reduction in stomatal aperture could be attributed to declining soil water availability as the 10 d cycle progressed. As soil moisture decreases, plants may respond by closing their stomata to conserve water, which in turn limits CO_2_ uptake and reduces photosynthetic rates (*A*_net_) (Ainsworth *et al.*, 2003). Conversely, non-stomatal limitation under e[CO_2_] may have played a role in the initial enhancement in *A*_net_. Analysis of the *A*–C_i_ curve did not show consistent effects indicative of a shift in the *A*–C_i_ response (C_i_×condition, *f*=0.87, *P*=0.35; C_i_×condition×CO_2_, *f*=3.5701, *P*=0.06; Supplementary Fig. S2). Although the latter interaction approached significance, suggesting subtle differences in the *A*–C_i_ response, a more detailed CO₂ response analysis—specifically, one investigating Rubisco kinetics—is needed to reveal further nuances. This result aligns with our main conclusion that stomatal regulation largely drives the response under these conditions. Plants under e[CO_2_] were able to maintain *g*_s_ as they probably experienced less water stress towards the end of the 10 d irrigation treatment than plants at ambient [CO_2_].

The observed changes in *g*_s_ and *A*_net_ led to changes in iWUE under e[CO_2_], in line with other experimental studies (Wohlfahrt *et al.*, 2018) and meta-analyses (Wang *et al.*, 2022). Notably, iWUE was enhanced by 50% under e[CO_2_] compared with ambient plots, greater than the expected proportional increase of 37.5% as hypothesized by optimal stomatal behaviour theory (Medlyn *et al.*, 2011a). The magnitude of the iWUE increase surpasses the enhancement due to the e[CO_2_] treatment, which prompts consideration of the underlying mechanisms (Medlyn *et al.*, 2011b). One plausible explanation could be that the increased *A*_net_ due to e[CO_2_] led to increased LAI, observed in November 2020. With a larger LAI, there would be more absorbed PAR, resulting in an amplified increase in iWUE (De Kauwe *et al.*, 2013). However, LAI was only enhanced under e[CO_2_] for 1 month, and for the remainder of the sampling period LAI was equal compared with ambient plots. Stomatal limitation may be another explanation for the increased iWUE under e[CO_2_]. Typically, under soil moisture stress, plants tend to prioritize water conservation, leading to a decrease in *g*_s_ (Flexas and Medrano, 2002). As a result, the ratio of *A*_net_ to *g*_s_ increases, and iWUE increases (De Kauwe *et al.*, 2013). This is because the plant can maintain or even increase *A*_net_ relative to the amount of water lost (Bacon, 2009). If the plant is already experiencing soil moisture stress, the increase in iWUE under e[CO_2_] might be more pronounced compared with conditions where soil moisture is not limiting. This is because stomatal limitation is often alleviated under e[CO_2_], leading to higher C_i_ and a greater increase in photosynthetic enhancement (Pathare *et al.*, 2017). However, there was little effect of irrigation frequency on the response of RSWC to e[CO_2_], and soil dry-down was slightly less under e[CO_2_] in the 5 d treatment (Fig. 3), given the reduction in *g*_s_. Additionally, there was no irrigation frequency effect on the response of iWUE to e[CO_2_]. Thus, we accept part of our first hypothesis that e[CO_2_] will enhance iWUE, by decreasing *g*_s_ and enhancing carbon assimilation, but reject that this difference will decrease as water availability decreases (i.e. irrigation frequency decreases). Further investigation into the interaction between [CO_2_] and water stress could provide insights into the dynamics of iWUE through stomatal limitation.

The steep decline in *g*_s_ in plants grown under ambient conditions in our study, coupled with water potential comparable with elevated plots, suggests that stomata may be regulating *g*_s_ through mechanisms such as abscisic acid (ABA) signalling or hydraulic feedback to maintain leaf water potential (Holloway-Phillips and Brodribb, 2011; Christmann *et al.*, 2013). These mechanisms enable the plant to close stomata in response to decreasing soil moisture, thereby stabilizing leaf water status (Schulze, 1986). Due to the already reduced stomatal aperture, plants growing at e[CO_2_] can maintain a more stable *g*_s_ as time passes after an application of water. The water conservation under e[CO_2_] may be sufficient to extend the period of enhanced photosynthesis (Owensby *et al.*, 1997) as reported above. Understanding how water use changes at the tree or whole-stand level resulting from variations in *g*_s_ at the leaf level is essential to account for various feedback effects (De Kauwe *et al.*, 2013). These encompass responses related to biomass and leaf area, along with factors influencing transpiration control (such as net radiation and atmospheric evaporative demand), and components of water balance (such as soil evaporation, run-off, and competition for water use) (De Kauwe *et al.*, 2021). Understanding this variation remains a critical gap in plant responses to e[CO_2_].

### Responses of LAI and soil water content

Understanding the response of LAI to e[CO_2_] is important for predicting crop production (Ewert, 2004); however, field studies show highly variable results (Kimball *et al.*, 2002; Ainsworth and Long, 2005). Studies have reported changes in LAI in *Lolium* spp., ranging from –15.7% to 12% under e[CO_2_] (Daepp *et al.*, 2001; Weigel and Manderscheid, 2012). Our study demonstrated that ryegrass LAI was strongly seasonal, with the greatest values occurring during spring. The stimulation of *A*_net_ under e[CO_2_] led to a 29% increase in LAI in November 2020 compared with ambient plots; however, for most of the study, LAI was unchanged. This highly seasonal response of LAI to e[CO_2_] has also been observed in rice, where the study reported that stimulation of LAI occurred early in the growing season while maximum LAI did not differ between treatments (Kim *et al.*, 2003). Enhanced LAI in November may be explained by particularly fast leaf elongation in spring that can be enhanced under e[CO_2_] (Schapendonk *et al.*, 1996). It is possible that phenological changes caused by e[CO_2_] (Cleland *et al.*, 2007) led to canopy closure in FACE plots earlier than in control plots, and LAI was maximal for the species at harvest. However, detailed data on leaf expansion at this site (Missen, 2023) indicate that this was not the case, and leaves kept growing right up until harvest. Additionally, there was also no change in LAI under the varied irrigation regimes, and these regimes did not influence the [CO_2_] effect on LAI (i.e. there was no [CO_2_]×irrigation interaction on LAI). Thus, our hypothesis that LAI would positively respond to e[CO_2_] compared with ambient conditions and that this difference would amplify with decreasing irrigation frequency is rejected.

Rather than changes in LAI under e[CO_2_], studies often report altered root:shoot ratios, suggesting a shift in functional attributes (Rogers *et al.*, 1995). However, Pritchard *et al.* (1999) noted that assessing total root:shoot ratios may be an insensitive indicator of what is happening to plant growth in terms of structure and function, and differences in root depth, branching, and morphology may be better indicators of water and nutrient uptake (Tremmel and Bazzaz, 1993). LAI response in this study was unlikely to have been driven by nutrient limitation since the experiment was supplied with relatively high applications of nutrients, and soil tests prior to establishment indicated that micronutrients were sufficient to support substantial growth (Brown, 2020). Bunce (2004) concluded that the lack of response of LAI to e[CO_2_] in many crop species suggests that LAI is not carbon limited under field conditions. This means that the source is more limiting than the sink, which is often seen in perennial species (Burnett *et al.*, 2016), and many studies have demonstrated that increasing source activity (i.e. a stimulation of photosynthesis) does not lead to increased yield (Long *et al.*, 2006; Ainsworth *et al.*, 2008).

The observed varying response of LAI to e[CO_2_] makes it difficult to draw conclusions on the role that vegetative structure has on SWC in this system. Decreased *g*_s_ and increased LAI under e[CO_2_] during spring may theoretically lead to comparable (with ambient) SWC. Decreased *g*_s_ and comparable LAI throughout the majority of the year may theoretically lead to increased SWC or decreased dry down under e[CO_2_] (∆SWC), most probably in the 5 d irrigation treatment from December to February (Fig. 3). This finding indicates an important time scale of influence over the role of irrigation change, stipulating when plants benefit most from the carbon fertilization effect; assimilating more carbon and transpiring less. Interestingly, summer Ψ_leaf_ was unchanged in any [CO_2_] or irrigation treatment, and did not respond to RSWC. This may signify enhanced rooting depth, with plants optimizing biomass allocation to access water more effectively (Iversen *et al.*, 2008; De Kauwe *et al.*, 2015), which both may lead to increased soil water availability (Li *et al.*, 2022). An earlier study at our experimental site showed that under e[CO_2_], plants increased allocation below-ground (Brown, 2020); however, roots are not easily measured, because they grow and die rapidly and unpredictably (Norby *et al.*, 2004; Burnett *et al.*, 2016). Thus, few studies have focused on plant water relation responses to changes in root production under e[CO_2_], with most focusing on nitrogen and carbon soil cycling (Iversen *et al.*, 2008; Franklin *et al.*, 2009). Therefore, it is not clear what the implications are of increased root biomass on plant water relations under e[CO_2_].

Observations suggest that in the 3 d irrigation treatment, plants see the direct benefits from the carbon fertilization effect by assimilating more carbon and losing less water through stomatal conductance in the days closely following irrigation. Assuming that e[CO_2_] lowers the overall water consumption of a plant in well-watered conditions, the difference in soil moisture content compared with ambient conditions will increase as soil water starts to decline (De Kauwe *et al.*, 2021); that is, soil under e[CO_2_] will be wetter than soil under ambient conditions. Once these stomatal savings induce sufficient soil moisture savings, plants then benefit from both increased assimilation and increased relative SWC. The possible biological reduction in amplitude of change in SWC in the 5 d irrigation treatment under e[CO_2_] compared with ambient plots suggests that in the days immediately following irrigation, the lower observed *g*_s_ was translating into soil water savings. Once this occurred, plants were benefitting from the direct effects (increased carbon assimilation) and indirect effects (soil moisture savings) of e[CO_2_]. In plots where precipitation events were less frequent, namely every 10 d, the faster decline in SWC under ambient [CO_2_] led to some stomatal closure, causing stomatal conductance rates in ambient conditions to equal that of plants growing under e[CO_2_] (Fig. 5D). Therefore, as days since precipitation increase, the difference in soil moisture content between ambient and elevated [CO_2_] lessens. Of course this depends on equal LAI and unchanged VPD (De Kauwe *et al.*, 2021), conditions that did occur for the majority of the study period at TasFACE2. It is possible that the water savings under e[CO_2_] in the 5 d irrigation treatment were reversed in the 10 d irrigation treatment, as towards the end of the irrigation cycle (7–10 d) there was no longer a reduction in *g*_s_; that is, stomata conductance was relatively higher under e[CO_2_] compared with ambient plots (which were experiencing more water limitation).

While ∆SWC allowed us to see short-term biological differences in soil moisture between [CO_2_] treatments, mean monthly RSWC was unable to detect the details of the cyclic pattern in soil moisture. It is possible that soil water potential (Ψ_soil_) would have been a better measure than RSWC, as small changes in SWC can equate to larger impacts on Ψ_soil_ (De Kauwe *et al.*, 2015). Measuring the temporal variability in SWC seems appropriate for determining the effects of e[CO_2_] on soil moisture and hence on plant physiological processes. The differences in treatment effects observed in ∆SWC and RSWC emphasize the difficulty in determining the direct and indirect effects of e[CO_2_] on plant physiology. We are unable to definitively accept or reject our third hypothesis that mean soil water content will be unchanged by e[CO_2_] due to an increase in LAI. This is because large temporal fluctuations in *g*_s_, SWC, and LAI were observed, challenging the simplistic assumption that the e[CO_2_] will reduce *g*_s_ and enhance yield. Our study demonstrates that irrigation variability, specifically reduced irrigation frequency, may see a decline in the expected water savings under e[CO_2_] while still maintaining increased carbon assimilation. This may be especially true for grasses, as changes in temporal rainfall strongly affect soil moisture variability at 0–30 cm (Volk *et al.*, 2000), where many species concentrate the majority of their roots (Houlbrooke *et al.*, 1997).

Tasmania’s future rainfall predictions are uncertain, with most model scenarios predicting lower annual rainfall into the future (Meyer *et al.*, 2021). Under a projected wetter scenario, more damaging and high-intensity rainfall would be expected (Meyer *et al.*, 2021). Under either scenario, Tasmania will see shifts in rainfall seasonality over the next 20–30 years. Autumn rainfall is projected to decrease significantly, with certain areas experiencing up to a 50% reduction compared with the 2010–2020 period. Additionally, there will be a consistent decline in spring rainfall, with some regions seeing 10–20% less rain and up to a 25% decrease by 2050 in the central north. Conversely, there will be a substantial increase in winter rainfall (Meyer *et al.*, 2021). Irrigation or rainfall frequency are thus likely to influence the CO_2_ effect in temperate pastures in Tasmania into the future, and a more nuanced understanding of these interactions is essential for accurately predicting the effects of e[CO_2_] on plant and ecosystem water dynamics.

### Conclusion

The water savings of plants under e[CO_2_] is complex and dynamic, and dependent on numerous factors. Here, we reported that e[CO_2_]-induced changes in *g*_s_ are a function of days since the last irrigation event. Our results clearly demonstrate that *g*_s_ declines over time to a greater extent in control plots than in e[CO_2_] plots, indicating that the relative advantage of e[CO_2_] in terms of carbon assimilation increases with time since irrigation. Thus, we believe the reason why the benefits of e[CO_2_] increase with increasing time since irrigation is simply that the *g*_s_ of plants growing at ambient [CO_2_] is more or less equal to that of plants growing at e[CO_2_] by 7–10 d after irrigation and, all else being equal, *A*_net_ will be greater at e[CO_2_] because C_i_ is also greater at e[CO_2_]. We also showed that e[CO_2_] enhanced iWUE and LAI; however, the increase in LAI occurred early in the growing season (spring), with LAI unchanged for the majority of the study period. Mean monthly RSWC did not respond to the observed decreases in *g*_s_; however, the amplitude of change of SWC was probably a better measure than mean monthly RSWC, and biologically this may have been greater for ambient plots in the 5 d irrigation treatment during summer, but high variation obscured statistical significance. The nature of the relationship between intra-annual rainfall variability and [CO_2_] has important implications for understanding the effects of climate change on perennial ryegrass, and its ability to provide agricultural production while supporting biodiversity and other ecosystem services. Future research should seek to explain how LAI and *g*_s_ interact to impact SWC, by investigating the timing of growth responses. Furthermore, examining seasonal precipitation variability which strongly influences soil moisture, may provide insight into plant responses to e[CO_2_].

## Supplementary data

The following supplementary data are available at *JXB* online.

Fig. S1. Experimental site map of TasFACE2 showing 12 experimental rings in a randomized split-plot design, with CO_2_ and irrigation treatments applied across sectors.

Fig. S2. Photosynthetic rate (*A*_net_) plotted against intercellular CO_2_ concentration (C_i_) under wet (1–6 d since irrigation) and dry (7–10 d since irrigation) conditions for ambient and elevated [CO_2_].

Table S1. Sampling days, replicates, and total samples taken of *A*_net_ and *g*_s_ in each treatment combination.

Table S2. Replicates and total samples taken of leaf water potential in each treatment combination at pre-dawn and midday.

erae511_suppl_Supplementary_Figures_S1-S2_Tables_S1-S2

## Data Availability

All data used in this study are available through the Dryad Digital Repository at DOI https://doi.org/10.5061/dryad.1c59zw40v (Missen *et al*., 2025). All statistical coding used in this study is available through Zenodo at DOI https://doi.org/10.5281/zenodo.11461965.
