# Supplementary material for: Increasing photosynthetic benefit with decreasing irrigation frequency in an Australian temperate pasture exposed to elevated carbon dioxide
Source: J Exp Bot. 2025 Apr 9;76(6):1795–810. doi: 10.1093/jxb/erae511 (PMC11981893; doi:10.1093/jxb/erae511)
Supplement: erae511_suppl_Supplementary_Figures_S1-S2_Tables_S1-S2 [file erae511_suppl_supplementary_figures_s1-s2_tables_s1-s2.pdf]

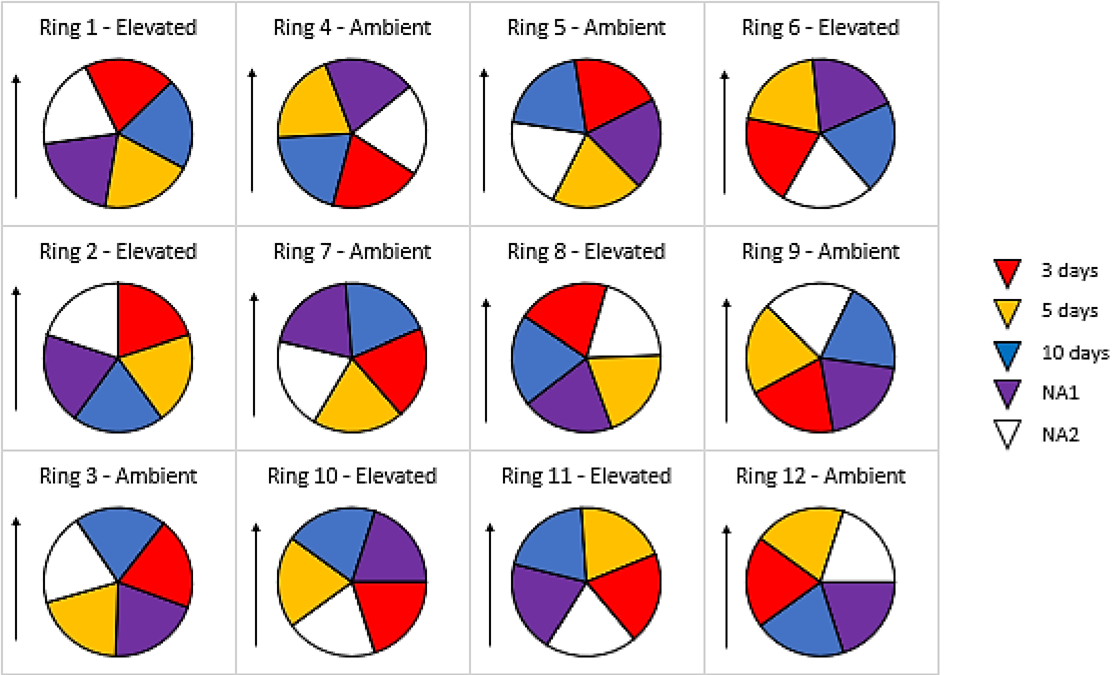

Figure S1. Experimental site map of TasFACE2 showing 12 experimental rings in a randomised split-plot design, with CO_2 and irrigation treatments applied across sectors.

Table S1. Sampling days, replicates and total samples taken of A_{net} and g_s in each treatment combination.

**[CO_2]**	**Irrigation**	**Rings sampled**	**Replicates**	**Sampling days in irrigation cycle**	**Total samples**
Ambient	3 days	3, 4, 5, 7, 9	5	1, 2, 3	41
Elevated	3 days	1, 2, 6, 8, 10	5	1, 2, 3	44
Ambient	5 days	3, 4, 5, 7, 9	5	1, 2, 3, 5	36
Elevated	5 days	1, 2, 6, 8, 10	5	1, 2, 3, 5	34
Ambient	10 days	3, 4, 5, 7, 9	5	2, 5, 7, 9, 10	54
Elevated	10 days	1, 2, 6, 8, 10	5	2, 5, 7, 9, 10	56

Table S2. Replicates and total samples taken of leaf water potential in each treatment combination at pre-dawn and midday.

[CO_2]	Irrigation	Time of day	Total samples	Replicates
Elevated	3 days	Pre-dawn	8	4
Ambient	3 days	Pre-dawn	8	4
Elevated	5 days	Pre-dawn	8	4
Ambient	5 days	Pre-dawn	9	4
Elevated	10 days	Pre-dawn	12	4
Ambient	10 days	Pre-dawn	12	4
Elevated	3 days	Midday	18	4
Ambient	3 days	Midday	16	4
Elevated	5 days	Midday	24	4
Ambient	5 days	Midday	23	4
Elevated	10 days	Midday	33	4
Ambient	10 days	Midday	26	4

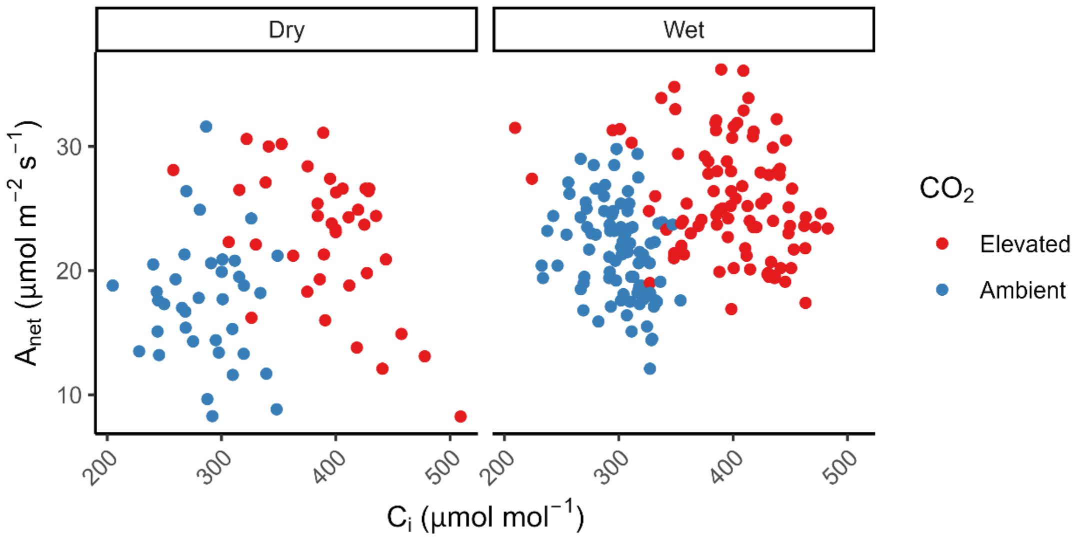

Figure S2. Photosynthetic rate (A_{net}) plotted against intercellular CO_2 concentration (C_i) under wet (1 – 6 days since irrigation) and dry (7 – 10 days since irrigation) conditions for ambient and elevated [CO_2].
